# Supplementary material for: COVID-19 vaccine coverage among immigrants and refugees in Alberta: A population-based cross-sectional study
Source: J Glob Health. 2022 Nov 7;12:05053. doi: 10.7189/jogh.12.05053 (PMC9725104; doi:10.7189/jogh.12.05053)
Supplement: Online Supplementary Document [file jogh-12-05053-s001.pdf]

ONLINE SUPPLEMENTARY DOCUMENT

Title: COVID-19 vaccine coverage among immigrants and refugees in Alberta: a population-based cross-sectional study

Authors: Shannon E MacDonald, Yuba Raj Paudel, Crystal Du

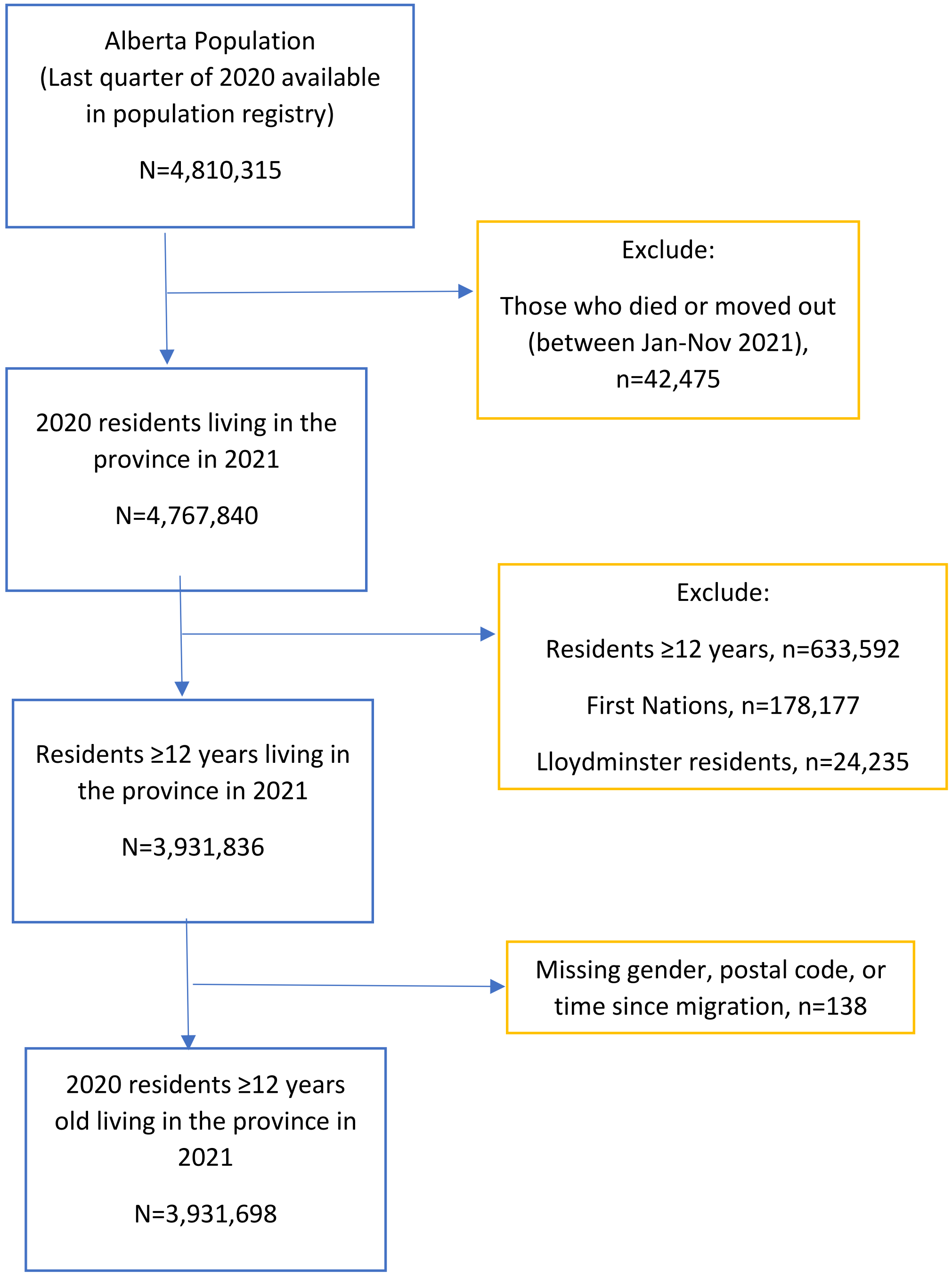

Figure S1. Flow diagram showing selection of participants.

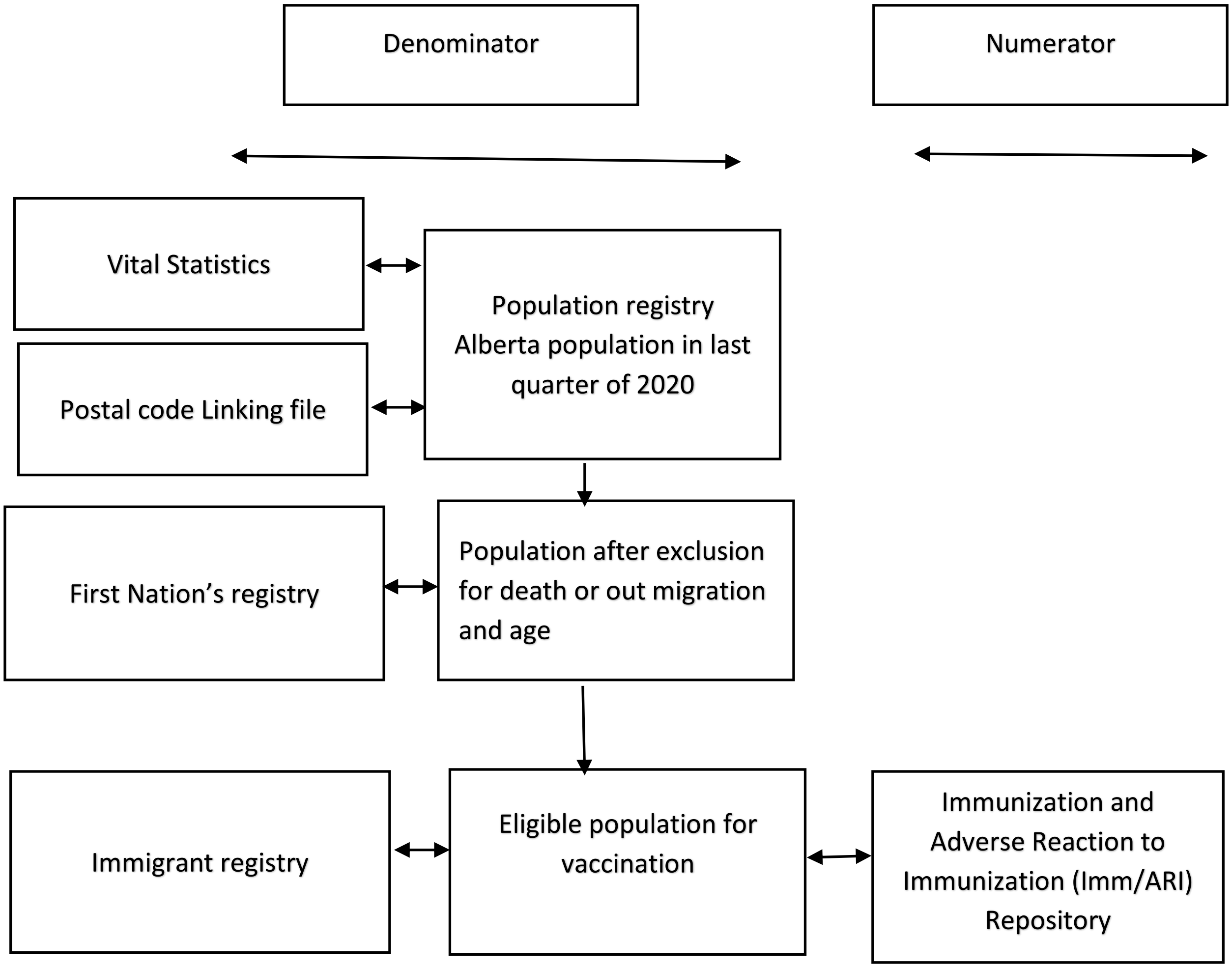

Figure S2. Linkage of different databases.

Table S1: Vaccination coverage of Albertan immigrants categorized by sub-continental region of origin

Continent	% (n)
North America	63.19% (43,439)
Meso America	62.42% (34,137)
South America	75.67% (17,299)
Europe Unspecified	72.45% (4,191)
East Europe	60.69% (11,512)
West Europe	64.05% (9,417)
North Europe	71.50% (24,356)
South Europe	70.34% (8,858)
Central Asia	64.59% (5,370)
Middle East	75.67% (41,263)
Africa Unspecified	79.70% (2,308)
North Africa	75.41% (7,825)
East Africa	79.51% (20,122)
Central/South Africa	76.41% (6,775)
West Africa	82.94% (14,112)
Asia Unspecified	86.21% (11,857)
East Asia	85.47% (193,589)
South Asia	83.71% (101,461)
Oceania	64.60% (7,700)
Unknown/Missing	80.16% (35,255)

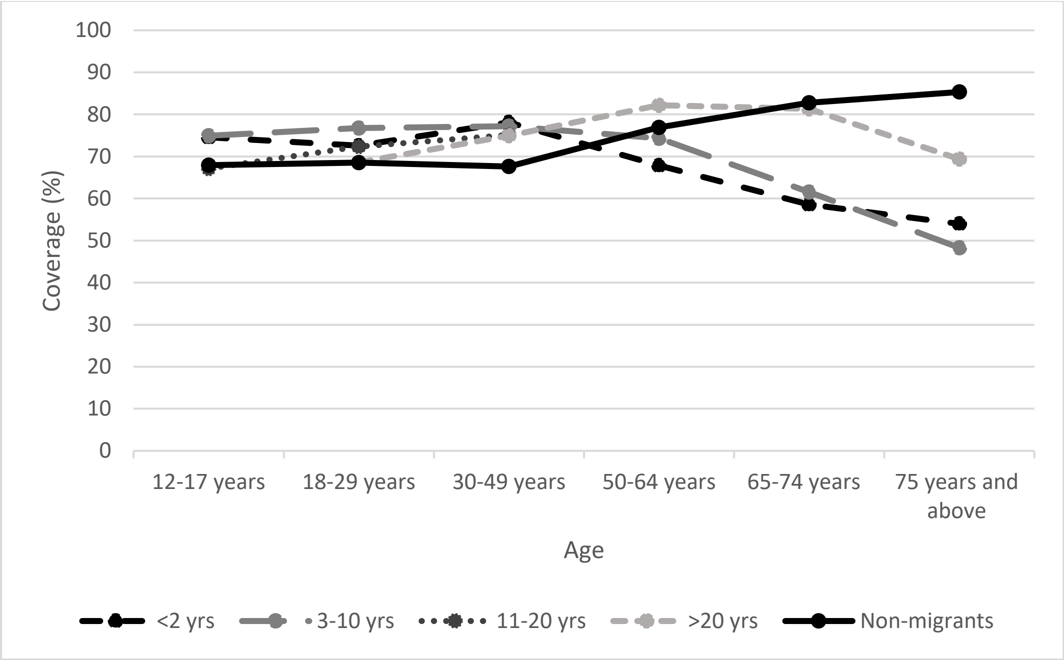

Figure S3. Vaccination coverage (2 doses) in different age categories by migration status.

Table S2. Time interval between two doses of COVID-19 vaccine (days)

Migration status	N	Mean (days)	Std Dev (days)	Median (days)
Immigrants (last 2 years)	44684	52	62	48
Immigrants (last 3-10 years)	219498	51	21	48
Immigrants (last 11-20 years)	182633	52	20	49
Immigrants (>20 years)	103301	55	21	53
Non-immigrants	2335331	54	28	51

Table S3: Proportion of Alberta residents who received no dose, one dose or two doses of a COVID-19 vaccine by migration status and sociodemographic characteristics.

Variables	Immigrants												Non-immigrants		
	Last 2 years			3-10 years			11-20 years			>20 years					
	No dose % (n)	One dose % (n)	Two dose % (n)	No dose % (n)	One dose % (n)	Two dose % (n)	No dose % (n)	One dose % (n)	Two dose % (n)	No dose % (n)	One dose % (n)	Two dose % (n)	No dose % (n)	One dose % (n)	Two dose % (n)
**Age**															
**12-17 years**	18.01 (779)	7.45 (322)	74.54 (3,224)	20.2 (5,930)	4.89 (1,434)	74.91 (21,988)	29.37 (3,523)	3.5 (420)	67.13 (8,051)	na	na	na	27.93 (68,763)	4.14 (10,202)	67.92 (167,212)
**18-29 years**	21.77 (4,553)	5.62 (1,175)	72.61 (15,184)	18.6 (12,136)	4.63 (3,021)	76.77 (50,081)	23.99 (9600)	3.69 (1,475)	72.33 (28,948)	27.86 (1,594)	3.55 (203)	68.59 (3,924)	26.54 (138,778)	4.88 (25,531)	68.58 (358,562)
**30-49 years**	17.8 (4,840)	4.1 (1,114)	78.11 (21,241)	19.63 (29,140)	3.18 (4,716)	77.19 (114,601)	22.26 (26,104)	2.63 (3,087)	75.11 (88,085)	22.38 (6,516)	2.72 (791)	74.9 (21,809)	29.06 (321,146)	3.33 (36,746)	67.61 (747,100)
**50-64 years**	29.19 (1,240)	2.92 (124)	67.89 (2,884)	23.46 (6,896)	2.23 (657)	74.31 (21,843)	21.48 (12,663)	1.78 (1,048)	76.74 (45,244)	15.93 (9,490)	1.88 (1,118)	82.19 (48,952)	20.87 (148,767)	2.25 (16,035)	76.88 (548,154)
**65-74 years**	38.24 (1,054)	3.16 (87)	58.6 (1,615)	36.5 (4,543)	1.93 (240)	61.57 (7,663)	27.25 (3,147)	1.84 (212)	70.92 (8,191)	17.34 (3,933)	1.33 (301)	81.34 (18,452)	15.81 (57,689)	1.37 (5,017)	82.81 (302,175)
**75 years & above**	43.81 (435)	2.22 (22)	53.98 (536)	50.26 (3,457)	1.44 (99)	48.3 (3,322)	44.09 (3,304)	1.0 (75)	54.9 (4,114)	29.44 (4,312)	1.17 (171)	69.39 (10,164)	13.69 (34,046)	0.98 (2,430)	85.33 (212,128)
**Sex**															
Female	21.37 (6,551)	4.62 (1,416)	74.01 (22,689)	20.73 (31,610)	3.37 (5,138)	75.9 (115,718)	22.77 (28,827)	2.38 (3,012)	74.86 (94,786)	18.48 (12,804)	1.83 (1,271)	79.69 (55,223)	22.25 (349,932)	2.58 (40,653)	75.17 (1,182,381)
Male	21.33 (6,350)	4..8 (1,428)	73.88 (21,995)	21.89 (30,492)	3.61 (5,029)	74.5 (103,780)	24.46 (29,514)	2.74 (3,305)	72.8 (87,847)	20.89 (13,041)	2.1 (1,313)	77.01 (48,078)	25.76 (419,257)	3.4 (55,308)	70.84 (1,152,950)
**Place of residence**															
Metro/ moderate Metro	19.19 (9,396)	4.66 (2,282)	76.15 (37,283)	20.54 (49,723)	3.47 (8,408)	75.99 (18,3997)	21.78 (45,321)	2.51 (5,225)	75.71 (157,53 5)	17.86 (20,014)	1.88 (2,102)	80.27 (89,959)	20.69 (439,843)	2.65 (56,242)	76.67 (163,0253)
Urban	23.57 (1,153)	4.93 (241)	71.5 (3,497)	20.75 (5,023)	3.98 (963)	75.27 (18,217)	24.62 (4,434)	2.87 (517)	72.51 (13,059)	24.57 (1,916)	2.32 (181)	73.11 (5,702)	28.81 (121,975)	3.6 (15,249)	67.58 (286,090)
Rural	35.76 (2,352)	4.88 (321)	59.36 (3,904)	28.92 (7,356)	3.13 (796)	67.95 (17,284)	40.5 (8,586)	2.71 (575)	56.79 (12,039)	33.02 (3,915)	2.54 (301)	64.44 (7,640)	31.86 (207,371)	3.76 (24,470)	64.38 (418,988)
**Income Quintile**															
Q1 (lowest)	19.85 (3,374)	6.09 (1,035)	74.06 (12,590)	20.65 (16,073)	4.57 (3,554)	74.78 (58,210)	25.12 (13,936)	3.14 (1,743)	71.73 (39,790)	20.03 (5,707)	2.27 (647)	77.7 (22,137)	28.58 (153,186)	3.87 (20,760)	67.55 (362,089)
Q2	21.88 (3,103)	4.36 (618)	73.76 (10,459)	20.34 (12,887)	3.48 (2,204)	76.19 (48,282)	24.4 (12,122)	2.6 (1,293)	72.99 (36,260)	19.62 (5,684)	1.94 (562)	78.44 (22,724)	25.8 (162,465)	3.19 (20,060)	71.02 (447,201)
Q3	21.78 (2,220)	4.27 (435)	73.96 (7,539)	21.39 (11,114)	3.16 (1,642)	75.45 (39,203)	23.52 (11,275)	2.33 (1,115)	74.15 (35,547)	19.77 (5,349)	1.88 (510)	78.35 (21,199)	23.62 (154,353)	2.87 (18,765)	73.51 (480,332)
Q4	21.98 (2,211)	4.01 (403)	74.02 (7,446)	21.76 (11,070)	2.99 (1,520)	75.26 (38,290)	22.41 (10,830)	2.37 (1,146)	75.21 (36,340)	19.23 (4,881)	1.8 (456)	78.97 (20,042)	22.37 (154,341)	2.73 (18,836)	74.89 (516,622)
Q5	22.15 (1,993)	3.92 (353)	73.92 (6,650)	22.96 (10,958)	2.61 (1,247)	74.42 (35,513)	22.18 (10,178)	2.22 (1,020)	75.6 (34,696)	19.35 (4,224)	1.87 (409)	78.78 (17,199)	20.95 (144,844)	2.54 (17,540)	76.52 (529,087)
